# Evaluation of ^18^F-AlF-NOTA-octreotide for imaging neuroendocrine neoplasms: comparison with ^68^Ga-DOTATATE PET/CT

**DOI:** 10.1186/s13550-021-00797-4

**Published:** 2021-06-09

**Authors:** Jiale Hou, Tingting Long, Zhiyou He, Ming Zhou, Nengan Yang, Dengming Chen, Shan Zeng, Shuo Hu

**Affiliations:** 1 Department of Nuclear Medicine, XiangYa Hospital, Central South University, No. 87 XiangYa Road, ChangSha, Hunan Province People’s Republic of China; 2 Department of Cancer Chemotherapy, XiangYa Hospital, Central South University, No. 87 XiangYa Road, ChangSha, Hunan Province People’s Republic of China; 3 Key Laboratory of Biological Nanotechnology, NHC. No. 87 XiangYa Road, ChangSha, 410013 Hunan Province People’s Republic of China; 4 National Clinical Research Center for Geriatric Disorders (XIANGYA), XiangYa Central South University, Changsha, 410008 Hunan People’s Republic of China

## Abstract

**Objective:**

To evaluate the diagnostic efficacy of ^18^F-AlF-NOTA-octreotide (^18^F-OC) PET/CT compared with that of ^68^Ga-DOTATATE PET/CT.

**Materials and methods:**

Twenty patients (mean age: 52.65 years, range: 24–70 years) with biopsy-proven neuroendocrine neoplasms (NENs) were enrolled in this prospective study. We compared the biodistribution profiles in normal organs based on the maximum standard uptake value (SUV_max_) and mean standard uptake value (SUV_mean_), and uptake in NEN lesions by measuring the SUV_max_ on ^18^F-OC and ^68^Ga-DOTATATE PET/CT images. The tumor-to-liver ratio (TLR) and tumor-to-spleen ratio were calculated by dividing the SUV_max_ of different tumor lesions by the SUV_mean_ of the liver and spleen, respectively. The Wilcoxon signed-rank test was used to compare nonparametric data. Data were expressed as the median (interquartile range).

**Results:**

In most organs, there were no significant differences in the biodistribution of ^68^Ga-DOTATATE and ^18^F-OC. ^18^F-OC had significantly lower uptake in the salivary glands and liver than ^68^Ga-DOTATATE. ^18^F-OC detected more lesions than ^68^Ga-DOTATATE. The uptake of ^18^F-OC in the tumors was higher in most patients, but the difference was not statistically significant relative to that of ^68^Ga-DOTATATE. However, the TLRs of ^18^F-OC were higher in most patients, including for lesions in the liver (*p* = 0.02) and lymph nodes (*p* = 0.02).

**Conclusion:**

Relative to ^68^Ga-DOTATATE, ^18^F-OC possesses favorable characteristics with similar image quality and satisfactory NEN lesion detection rates, especially in the liver due to its low background uptake. ^18^F-OC therefore offers a promising clinical alternative for ^68^Ga-DOTATATE.

**Supplementary Information:**

The online version contains supplementary material available at 10.1186/s13550-021-00797-4.

## Introduction

Neuroendocrine neoplasms (NENs) are a relatively rare and highly heterogeneous tumor derived from neuroendocrine cells. The incidence and prevalence of NENs have increased steadily over the past 40 years, with increasing awareness and emergence of better diagnostic tools [1]. This has been accompanied by a concomitant increase in the rate of NEN distant metastases, which negatively affects NEN treatment and survival [2]. Thus, effective NEN monitoring using sensitive imaging approaches is needed to detect progression and adapt treatment strategies.

NENs commonly express somatostatin receptors (SSTRs), making them amenable to molecular imaging with radionuclide-coupled somatostatin analogs as a diagnostic tool [3]. Currently, ^68^Ga-labeled somatostatin analogs (SSAs) for positron emission tomography/computed tomography (PET/CT) have been used in routine clinical practice [4, 5]. Relative to single-photon emission computed tomography (SPECT), PET has a higher spatial resolution, shorter imaging times, lower radiation exposure, and better lesion detection [6, 7]. Thus, PET scanning using ^68^Ga-labeled SSAs is critical for tumor detection rate, staging and restaging and post-therapy follow-up [4]. However, the use of ^68^Ga-labeled PET is limited by the high cost of ^68^Ge/^68^Ga generators [8] and the relatively short half-life of ^68^Ga (68 min). ^18^F-labeled (^18^F half-life: 106.9 min) SSAs have high tumor-to-background ratio (TBR) for NEN lesions; thus, these probes may be used as an alternative in NEN imaging and also allow for longer transport times [9, 10].

In recent years, there has been increased research on ^18^F-labeled agents [10–12]. ^18^F-AlF-NOTA-octreotide (^18^F-OC) exhibits satisfactory biodistribution and dosimetry profiles with a high NEN lesion detection rate [13]. A comparison of the imaging parameters of ^18^F-OC and ^68^Ga-DOTATATE for NENs in a small number of patients found that ^18^F-OC has excellent dynamics and imaging characteristics [14]. Here, we assessed the clinical applicability and efficacy of ^18^F-OC relative to those of ^68^Ga-DOTATATE in a larger group of patients.

## Methods

### *Patients and study design*

The research was approved by the institutional ethics review committee of Xiangya Hospital, Central South University for research purposes only (No. 20181001). All study participants gave written informed consent before the start of the study. Patients with clinically confirmed NENs were prospectively recruited into the study. Participants received an intravenous injection of ^18^F-OC and ^68^Ga-DOTATATE for PET/CT within 8 days (range: 1–8 days), except patients No. 1 (interval time: approximately 147 days without any treatment) and No. 15 (interval time: approximately 279 days without peptide receptor-radionuclide therapy (PRRT)).

### ^***68***^***Ga-DOTATATE and ***^***18***^***F-OC preparation***

^68^Ga-DOTATATE was synthesized using the acetone method on a fully automated Modular Lab system (Eckert & Ziegler, Germany), and quality control was performed as previously described [15]. The radiochemical purity of ^68^Ga-DOTATATE was > 90%. ^18^F-OC was produced as previously described [16] and under good manufacturing practice guidelines.

### *PET/CT image acquisition*

The study was carried out with a General Electric PET/CT scanner (Discovery 690 Elite, General Electric Health care, Waukesha, Wis). ^18^F-OC PET/CT imaging was performed 60 min after the radiotracer was intravenously (IV) injected at a dose of 3.7–4.44 MBq (0.1–0.12 mCi) per kilogram of body weight. ^68^Ga-DOTATATE imaging was performed 50 min after an injection with a total activity of 194.4 ± 37.9 MBq. First, a low-dose CT scan (120 kV; automatic mAs; pitch, 1:1; slice thickness, 3.75 mm; matrix, 512 × 512) was performed from the head to mid-thigh for anatomical localization and attenuation correction. Next, PET scanning was performed, with 2 min per bed position. Finally, images were reconstructed using the 3-dimensional ordered-subsets expectation maximization algorithm with 2 iterations and 23 subsets.

### *Image analysis*

Regions of interest (ROIs) were drawn on fused PET/CT images on a dedicated nuclear medicine AW 4.6 workstation (General Electric Healthcare) to obtain standardized uptake values (SUVs). ^18^F-OC and ^68^Ga-DOTATATE images were independently assessed by 2 experienced nuclear medicine physicians who were blinded to the patients and their medical information. The ROIs for measuring the maximum standard uptake value (SUV_max_) and mean standard uptake value (SUV_mean_) in normal organs and tissues and the ROIs for measuring the SUV_max_ of NEN lesions were drawn on serial images. The mean SUV_max_ and SUV_mean_ in the reference organs were evaluated by placing 3 consecutive ROIs (including the area with the highest uptake and that on the upper and lower slices based on visual assessment) inside the organ of interest, including pituitary, cerebral cortex, adrenal gland, uncinate process of the pancreas (PU), pancreas (except the PU), stomach, spleen, thyroid, salivary glands, liver, bone, renal parenchyma, small intestine, uterus (female), prostate (male), colon, lung, fat, myocardium, muscle, bladder wall, and blood pool, on both scans. Candidate lesions with activities greater than the physiologic uptake in the involved organs were considered lesions. These lesions were divided into 5 regions or groups: primary tumor, liver metastases, bone metastases, lymph node metastases, and metastases in other organs (lung, muscle, stomach, rectum, peritoneum, soft tissue, and thyroid). For patients with multiple lesions, at most 5 lesions with the highest uptake per organ were included in the uptake analysis. The tumor-to-liver ratio (TLR) and tumor-to-spleen ratio (TSR) were calculated by dividing the SUV_max_ of different tumor lesions by the SUV_mean_ of the liver and spleen in each patients, respectively. All ratios on corresponding ^18^F-OC and ^68^Ga-DOTATATE scans were computed from the same layer on the 2 scans. All discrepant lesions between the images of the 2 radiotracers were identified by other imaging or patient follow-up (computed tomography (CT), magnetic resonance imaging (MRI), and PET/CT) and then classified as true- or false-positive findings.

### *Statistical analysis*

Data analysis was performed using GraphPad Prism 6 (Version 6.01, 2012). Data are expressed as the median (interquartile range). Nonparametric data were compared using the Wilcoxon signed-rank test. *P* < 0.05 indicates statistical significance.

## Results

**Fig. 1:**
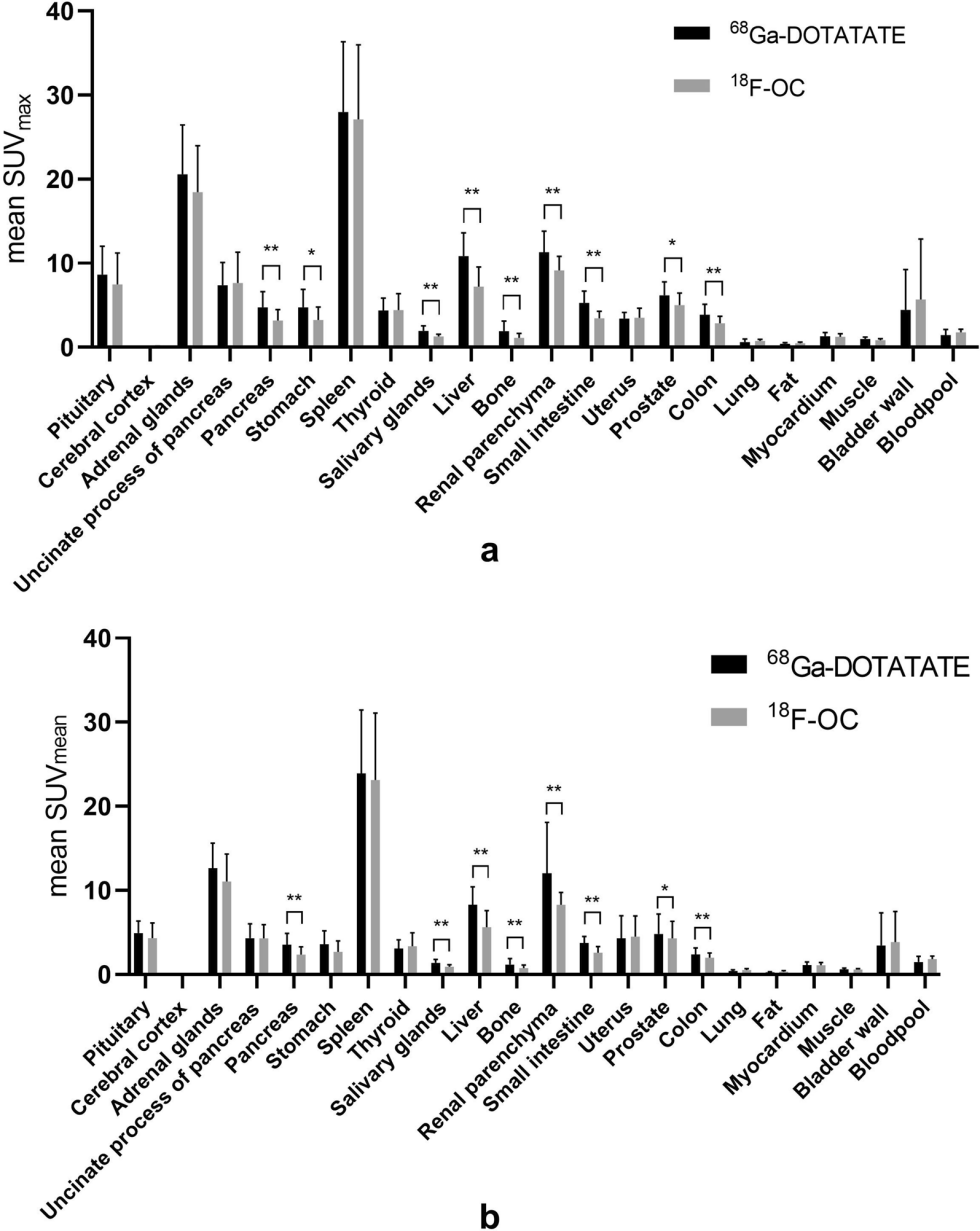
Uptake of ^68^Ga-DOTATATE and ^18^F-OC in normal organs was calculated in patients based on the mean SUV_max_ (**a**) and SUV_mean_ (**b**). Significant differences between ^68^Ga-DOTATATE and ^18^F-OC are indicated. ^**^: *p* =  < 0.01, ^*^: *p* =  < 0.05

Twenty patients were prospectively enrolled in the study, and their clinical characteristics are summarized in Table 1. No patients received PRRT treatment between ^68^Ga-DOTATATE and ^18^F-OC PET/CT scans. Both radiotracers were tolerated well by all patients, and no adverse events were reported. The physiological uptake of ^68^Ga-DOTATATE and ^18^F-OC is shown in Fig. 1. ^68^Ga-DOTATATE and ^18^F-OC PET/CT scans were compared at the lesion and region levels and based on SUV.

**Table 1:** Patient clinical characteristics

Patient (No)	Age (y)	Gender	Primary tumor	Biopsy site	Tumor grade	Ki67 value	Indication of imagine	Primary tumor resected	Time between scans (days)
1	55	M	Throat	Throat	1	2	Restage	Yes	147
2	52	M	Ileocecus	Ileocecus	2	NA	Restage	Yes	2
3	63	M	Rectum	Rectum	2	3	Restage	Yes	5
4	42	F	Rectum	Rectum	1	1	Stage	No	2
5	24	F	Pancreas	Liver	3	40	Restage	No	4
6	65	M	Stomach	Stomach	3	70	Stage	No	4
7	57	M	Pancreas	Pancreas	1	1	Stage	No	2
8	29	F	Unknown	liver	2	8	Stage	No	1
9	48	F	Rectum	Rectum	2	NA	Restage	Yes	5
10	70	F	Small intestine	Small intestine	1	NA	Restage	Yes	8
11	67	M	Pancreas	Liver	2	NA	Stage	No	7
12	68	M	Pancreas	Liver	1	< 2	Stage	No	5
13	59	M	Unknown	Liver	1	1	Stage	No	1
14	60	F	Pancreas	Pancreas	1	1	Stage	No	1
15	46	F	Unknown	Liver and Celiac	1	1	Stage	No	279
16	56	F	Pancreas	Pancreas	Insulinoma		Stage	No	1
17	57	M	Unknown	Neck	2	15	Stage	No	1
18	50	M	Pancreas	Pancreas	2	10	Stage	No	1
19	49	M	Rectum	Rectum	2	5	Restage	Yes	2
20	47	F	Paraganglioma	Retroperitoneum	NA	NA	Stage	No	1

*NA* not applicable

### ***Biodistribution of ***^***68***^***Ga-DOTATATE and ***^***18***^***F-OC***

Similar to that of ^68^Ga-DOTATATE, the highest SUV_max_ values for ^18^F-OC were recorded in the spleen, adrenal gland, renal parenchyma, pituitary gland, liver, and PU. Lower SUV_max_ and SUV_mean_ values were observed in the salivary glands, myocardium, bone, lung muscle, fat, and cerebral cortex. In most organs, the biodistribution of ^68^Ga-DOTATATE was not significantly different from that of ^18^F-OC. Relative to ^68^Ga-DOTATATE, ^18^F-OC had significantly lower uptake in organs such as the salivary glands, liver, pancreas, bone, renal parenchyma, and prostate (Fig. 1).

### ***Comparison of tumor detection rates between ***^***68***^***Ga-DOTATATE and ***^***18***^***F-OC PET/CT***

This study included 20 NEN patients. Examinations using ^68^Ga-DOTATATE and ^18^F-OC PET/CT revealed that 19 patients had lesions and 1 patient had no lesions. Follow-up examination confirmed lesions in the 19 patients and that patient No. 1 had no lesions. Table 2 shows the discordant lesions examined by ^68^Ga-DOTATATE and ^18^F-OC PET/CT.

**Table 2:** Patients with discordant lesions on ^18^F-OC and ^68^Ga-DOTATATE PET/CT

Patient	Primary tumor		Liver metastases		Bone metastases		Lymph node metastases		Other sites metastases		Total lesions	
	^68^Ga-DOTATATE	^18^F-OC	^68^Ga-DOTATATE	^18^F-OC	^68^Ga-DOTATATE	^18^F-OC	^68^Ga-DOTATATE	^18^F-OC	^68^Ga-DOTATATE	^18^F-OC	^68^Ga-DOTATATE	^18^F-OC
3	–	–	0	1	–	–	–	–	–	–	0	1
4	1	1	–	–	–	–	1	2	1	1	3	4
5	–	–	–	–	5	5	3	2	–	–	8	7
9	–	–	1	1	3	3	1	1	7	10	12	15
10	–	–	1	2	–	–	3	3	–	–	4	5
13	–	–	9	12	–	–	3	3	–	–	12	15
14	1	1	9	19	–	–	2	2	–	–	12	22
18	1	1	10	18	–	–	–	–	–	–	11	19
19	–	–	–	–	–	–	1	2	–	–	1	2
20	–	–	–	–	–	–	–	–	4	3	4	3

**Fig. 2:**
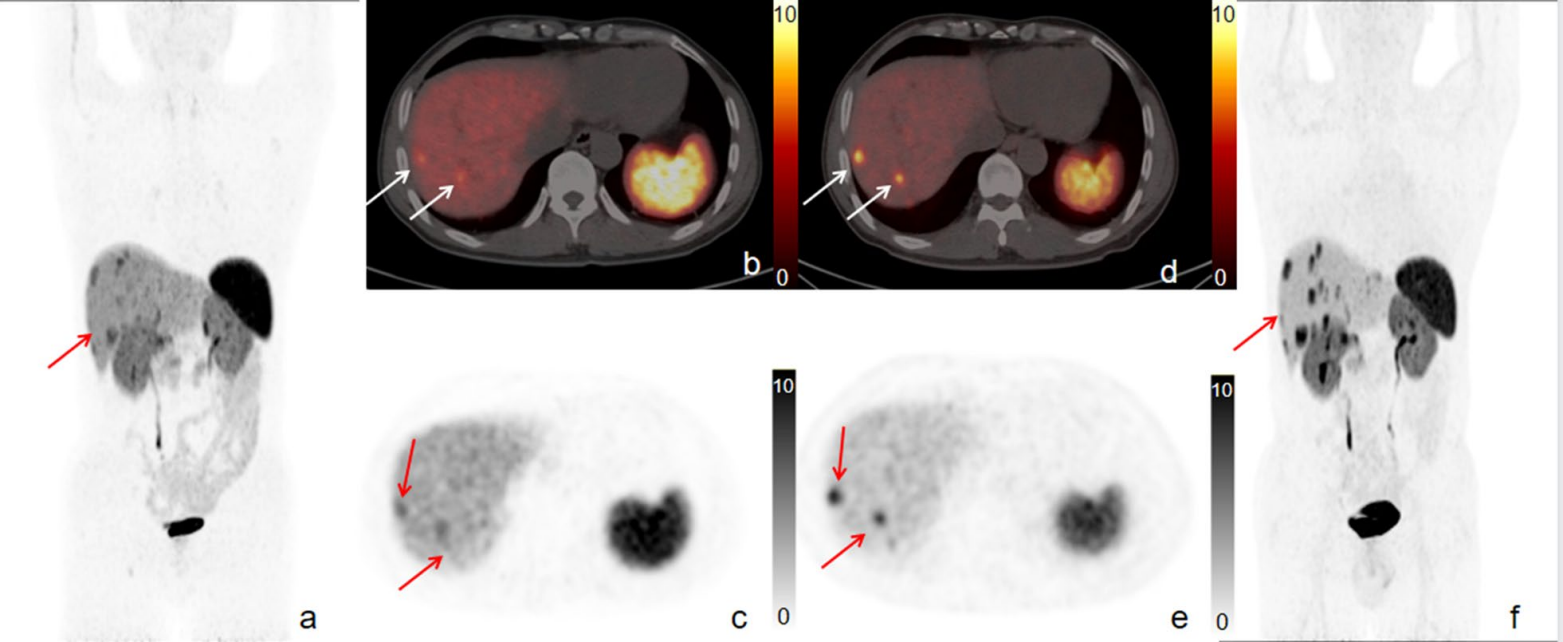
More liver lesions were detected by ^18^F-OC (**d–f**) than by ^68^Ga-DOTATATE (**a**–**c**). Maximum intensity projection image (**a**, **f**) shows more liver metastases present in a 50-year-old patient with a grade II primary pancreatic neuroendocrine tumor. Although transaxial fused PET/CT (**b**, **d**) and PET images (**c**, **e**) acquired with ^68^Ga-DOTATATE and ^18^F-OC show an equal number of liver lesions in this slice, the lower background level of ^18^F-OC uptake by normal liver (**d**–**f**) better delineates liver lesions by making the lesions appear to have more obvious uptake and sharper edges relative to background levels of ^68^Ga-DOTATATE uptake (**a**–**c**)

In the region-based comparison, 9 patients had primary tumors on both ^18^F-OC and ^68^Ga-DOTATATE images. In addition, there were 4 patients staged with unknown primary lesions. Sixteen patients had metastases on ^68^Ga-DOTATATE PET/CT, and 17 patients had metastases on ^18^F-OC PET/CT. ^18^F-OC demonstrated a higher ability to detect liver lesions (Fig. 2). In 11 patients with liver metastases, 100% (11/11) and 90.9% (10/11) of patients showed liver metastases on ^18^F-OC and ^68^Ga-DOTATATE scans, respectively. ^18^F-OC also detected peritoneal lesions more effectively than ^68^Ga-DOTATATE in 1 patient (No. 9).

**Fig. 3:**
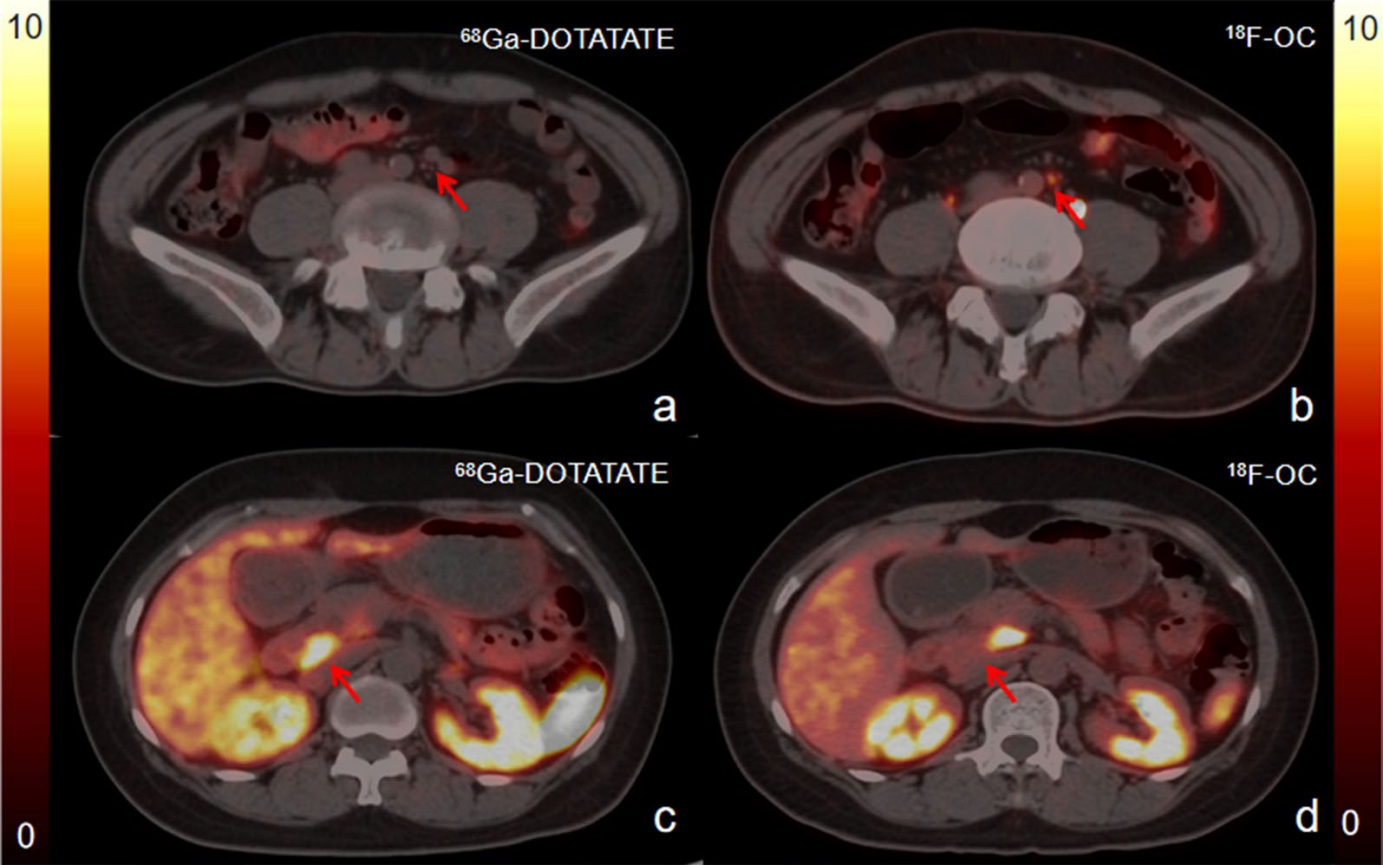
Both ^18^F-OC and ^68^Ga-DOTATATE had lesions that could not be detected by another imaging agent. PET/CT images acquired with ^68^Ga-DOTATATE and ^18^F-OC for patient No. 19 (**a**, **b**) and No. 20 (**c**, **d**). The ^18^F-OC fusion image (**b**) found an increased uptake in a retroperitoneal lymph node, while the uptake of this lymph node was not significantly increased on ^68^Ga-DOTATATE (**a**) image. However, in another patient, the ^18^F-OC fusion image (**d**) shows that the uptake in one retroperitoneal lesion was not obvious, while the uptake of ^68^Ga-DOTATAE was significant (**c**)

In the lesion-based examination, ^68^Ga-DOTATATE and ^18^F-OC PET/CT detected 152 and 177 focal lesions, respectively (*p* = 0.54). A total of 149 tumor lesions (9 in the primary sites, 93 in the liver, 20 in the lymph node, 8 in the bone, and 19 in other sites) were concordantly detected on both ^18^F-OC and ^68^Ga-DOTATATE PET/CT scans. An additional 30 lesions were detected by one of the scans only (Table 2). Both ^18^F-OC and ^68^Ga-DOTATATE had lesions that could not be detected by another imaging agent (Fig. 3). ^18^F-OC detected 28 lesions (23 in the liver, 2 in the lymph node, and 3 in the peritoneum) not visualized with ^68^Ga-DOTATATE. ^68^Ga-DOTATATE identified 1 lymph node lesion and 1 retroperitoneal lesion not seen with ^18^F-OC. ^18^F-OC detected significantly more liver lesions (116 vs. 93, *p* < 0.01). There was a difference of 10 liver metastases detected by the 2 radiotracers in patient No. 14 (Fig. 2), which were confirmed as true lesions by follow-up CT and MR. Additionally, ^18^F-OC detected 3 peritoneal lesions in patient No. 9. Regarding lymph node lesions, both ^18^F-OC and ^68^Ga-DOTATATE detected 1 lesion that was not clearly detected by the other imaging agent. In addition, ^18^F-OC and ^68^Ga-DOTATATE PET/CT had comparable effectiveness in detecting primary tumors and bone metastases.

**Fig. 4:**
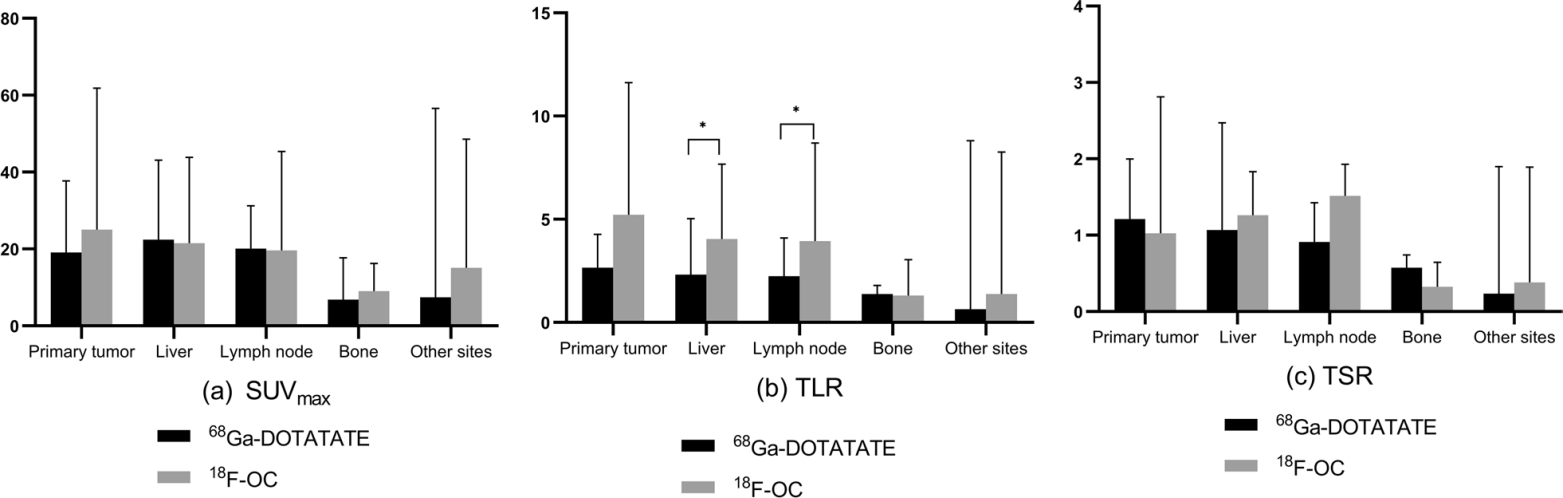
Bar chart representing the maximum standardized uptake value (SUV_max,_ **a**), tumor-to-liver ratio (TLR, **b**) and tumor-to-spleen ratio (TSR, **c**) of ^18^F-OC. TLR (**b**) and TSR (**c**) were calculated by dividing the SUV_max_ of tumor lesions by the patient-specific SUV_mean_ of the liver and spleen, respectively. The TLRs for lesions in the liver and lymph nodes were significantly higher with ^18^F-OC (*p* = 0.02 and *p* = 0.02, respectively). All other SUV_max_, TLR and TLR calculations did not show significant differences

Lesion uptake analysis found that ^18^F-OC uptake was slightly higher than ^68^Ga-DOTATATE uptake in primary tumors and metastases, but there were no significant differences (primary tumor: 25.01 (16.52–38.58) versus 19.08 (16.37–34.28), *p* = 0.80; metastases: 18.24 (11.25–37.48) versus 17.13 (8.99–28.72), *p* = 0.33). However, some lesions had higher ^18^F-OC uptake and others lesions had higher ^68^Ga-DOTATATE uptake, even among lesions of the same patient. For example, on PET/CT for suspected retroperitoneal pheochromocytoma in patient No. 20, 3 lesions were seen in the neck region, with higher ^68^Ga-DOTATATE uptake than ^18^F-OC uptake in 2 of the neck lesions (SUV_max_ 56.59 vs. 53.11 and 27.19 vs. 15.13), but higher ^18^F-OC uptake in the other lesion (SUV_max_ 115.14 vs. 111.45) (Additional file 1: Fig. S1). Furthermore, in liver and lymph node lesions, the ^18^F-OC TLR was higher than that with ^68^Ga-DOTATATE (*p* = 0.02, Fig. 4). However, the ^18^F-OC TSR was not significantly higher than that of ^68^Ga-DOTATATE for primary tumor or metastases (Fig. 4).

In our study, we found that despite physiological uptake in the PU (mentioned above), three cases of nodules with abnormal density or signal in the PU on CT or MRI showed abnormal uptake in the PU on ^68^Ga-DOTATATE and ^18^F-OC PET (Additional file 2: Fig. S2). The SUV_max_ were as follows: 41.1, 86.7, 16.4, respectively in ^68^Ga-DOTATATE and 27.5, 94.3, 15.3, respectively in ^18^F-OC.

## Discussion

Here, we prospectively assessed the performance of ^18^F-OC PET/CT relative to ^68^Ga-DOTATATE PET/CT in 20 NEN patients. The ^18^F-OC had a favorable biodistribution profile and was not inferior to ^68^Ga-DOTATATE in tumor uptake, TLR and TSR.

Our data showed that the ^18^F-OC distribution in organs was similar to that of ^68^Ga-DOTATATE. ^18^F-OC accumulation was very high in the spleen, which was similar to that of ^68^Ga-labeled DOTA-SSAs. Because both radiotracers were mainly excreted by the urinary system, higher uptake was seen in the kidneys. However, the overall uptake of ^18^F-OC in organs was lower than that of ^68^Ga-DOTATATE, especially in the liver, where the background ^68^Ga-DOTATATE uptake was 1.5 times greater than that of ^18^F-OC. We found that the salivary glands showed visible differences between the 2 radiotracers, which was consistent with past findings that ^68^Ga-DOTATATE uptake by salivary glands was four–sixfold higher than that of ^18^F-OC, mainly because of different radiotracer clearance times [14].

Because of high physiological uptake due to high SSTR2 expression in the PU and artifacts caused by respiratory movement, focal pancreatic lesions and lesions around the head of the pancreas may be obscured. Here, we found that both ^18^F-OC and ^68^Ga-DOTATATE had high uptake nodules in the PU, and other imaging examinations (CT or MRI) showed changes in the shape, signal or density of these nodules (Additional file 2: Fig. S2). Other imaging agents based on ^68^Ga-labeled radionuclides also demonstrated high sensitivity and specificity for detecting lesions (93.6% and 90%, respectively) in the PU [17]. We considered that results of a previous study [17] and ours could indicate SSA- PET, combined with morphological information (CT or MRI), especially if performed with enhanced CT or MRI, will improve the accuracy of lesions in the PU. But our number of cases was relatively small (*n* = 3). Thus, larger studies are needed to confirm these findings.

^18^F-OC and ^68^Ga-DOTATATE were highly sensitive in detecting lesions, and there were no differences in their overall diagnostic efficacy. Relative to ^68^Ga-DOTATATE, ^18^F-OC can detect lesions better (177 vs. 152), especially lesions in the liver (116 vs. 93), probably due to the lower background level of ^18^F-OC uptake in the liver. This finding is of great clinical significance, as it may affect treatment methods. For example, in patient No. 3, liver lesions were detected with ^18^F-OC but not ^68^Ga-DOTATATE. In patient No. 10, only one lesion was detected in the left lobe with ^68^Ga-DOTATATE, while ^18^F-OC detected another lesion in the right lobe of the liver, which was confirmed to be NEN metastases through pathology. These data are consistent with findings by Pauwels et al. [14] that ^18^F-OC detects more liver lesions.

Our data did not uncover differences between ^68^Ga-DOTATATE and ^18^F-OC in the detection of bone lesions (8 vs. 8). However, Pauwels et al. [14] found that ^18^F-OC detects more bone lesions. The differences between the 2 studies may be due to the small number of bone lesions in our study. Regarding lymph node lesions, both imaging radiotracers detected unique lesions. Additionally, ^18^F-OC detected 3 relatively small peritoneal metastases (diameter: < 5 mm) in patient No. 9, which were missed by ^68^Ga-DOTATATE. This is attributable to ^18^F being a typical short-distance positron emitter with better spatial resolution [18], which may be better suited for detecting small lesions. Thus, the capacity of ^18^F-OC to detect lesions is similar to that of ^68^Ga-DOTATATE, and ^18^F-OC may detect liver lesions more efficiently.

In this study, the SUV_max_ of ^18^F-OC was higher than that of ^68^Ga-DOTATATE, but the difference was not statistically significant. Interestingly, relative to ^68^Ga-DOTATATE, ^18^F-OC had a better target-to-background ratio. In this study, using liver and spleen for background comparisons, we found the ^18^F-OC TLRs for lesions in the liver and lymph node were significantly higher than those of ^68^Ga-DOTATATE, probably due to low liver background with ^18^F-OC. However, this finding differs from the results from Pauwels et al. [14] that the ^18^F-OC SUV_max_ for all lesions were significantly lower than those of ^68^Ga-DOTATATE, but there was no difference in TBR, which may be attributable to the small sample size. We also found that some patients exhibited higher ^68^Ga-DOTATATE uptake in lesions while others had higher ^18^F-OC uptake, and even within the same patient, some lesions had higher ^68^Ga-DOTATATE uptake while others had greater ^18^F-OC uptake; this is probably because of NEN heterogeneity [19]. The reason for the difference between the two radiotracers still needs further study.

Taken together, we found that ^18^F-OC had similar characteristics to ^68^Ga-DOTATATE in terms of physiological distribution, lesion detection, and lesion uptake. However, ^18^F-OC was relatively better in detecting liver lesions than ^68^Ga-DOTATATE. The two radiotracers had significantly difference TLRs, which is an important parameter for lesion detection.

### Limitations

The most significant limitation of this study was the lack of pathological confirmation of most lesions, which was not performed due to the ethical implications of pathologically examining all patient lesions. Thus, all lesions found with ^18^F-OC and ^68^Ga-DOTATATE were confirmed using alternative imaging approaches such as ^18^F-FDG PET, CT, or MRI. In addition, due to the small size of the study group and the small number of patients with higher-grade NENs, we could not evaluate the correlation between uptake and tumor grade. Future studies will involve a larger sample size.

## Conclusion

Overall, ^18^F-OC shows a favorable biodistribution, in which the uptake in various organs is similar to or even lower than that of ^68^Ga-DOTATATE. ^18^F-OC can detect liver lesions better than ^68^Ga-DOTATATE, with a better tumor-to-liver ratio. However, both ^18^F-OC and ^68^Ga-DOTATATE have similar detection rates for lesions in other organs. In general, ^18^F-OC has great potential as an alternative to ^68^Ga-DOTATATE in the absence of a ^68^Ge/^68^Ga generator. In the future, more patients are needed for comparisons between ^68^Ga-DOTATATE and ^18^F-OC to verify the value of ^18^F-OC in clinical applications.

## Supplementary Information

**Additional file 1.**. Inconsistent uptake of 68Ga-DOTATATE (a–c) and 18F-OC in different lesions of a patient.

**Additional file 2.**. The lesions of ^68^Ga-DOTATATE (a–c) and F-OC in the uncinate process of the pancreas (PU) of two patients.
