# Supplementary material for: Evaluation of 18F-AlF-NOTA-octreotide for imaging neuroendocrine neoplasms: comparison with 68Ga-DOTATATE PET/CT
Source: EJNMMI Res. 2021 Jun 9;11:55. doi: 10.1186/s13550-021-00797-4 (PMC8190415; doi:10.1186/s13550-021-00797-4)
Supplement: Supplementary file 1 — Additional file 1.. Inconsistent uptake of 68Ga-DOTATATE (a–c) and 18F-OC in different lesions of a patient. [file 13550_2021_797_MOESM1_ESM.docx]

**Additional file 1: Figure S1**

**
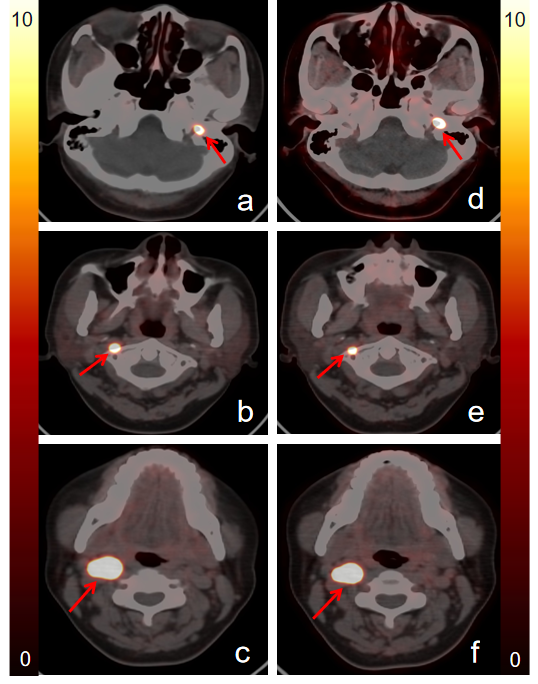
**

A patient who suspected paraganglioma underwent ^68^Ga-DOTATATE (a-c) and ^18^F-OC (d-f). Fusion PET/CT images found 3 other lesions (red arrows) in the area of neck. ^68^Ga-DOTATATE (a-c) and ^18^F-OC (d-f) PET/CT images revealed intense tracer uptake in the lesions suggesting SSTR receptor expression. However, the ^68^Ga-DOTATATE uptake of the two neck lesions (a, b, d, e) are higher than that of ^18^F-OC (SUV_max_ 56.59 vs. 53.11 and 27.19 vs. 15.13), and another lesion in the neck (c, f) has higher ^18^F-OC uptake than ^68^Ga-DOTATATE (SUV_max_ 115.14 vs 111.45).
