# Supplementary material for: Evaluation of 18F-AlF-NOTA-octreotide for imaging neuroendocrine neoplasms: comparison with 68Ga-DOTATATE PET/CT
Source: EJNMMI Res. 2021 Jun 9;11:55. doi: 10.1186/s13550-021-00797-4 (PMC8190415; doi:10.1186/s13550-021-00797-4)
Supplement: Supplementary file 2 — Additional file 2.. The lesions of 68Ga-DOTATATE (a–c) and F-OC in the uncinate process of the pancreas (PU) of two patients. [file 13550_2021_797_MOESM2_ESM.docx]

**Additional file 2: Figure S2**


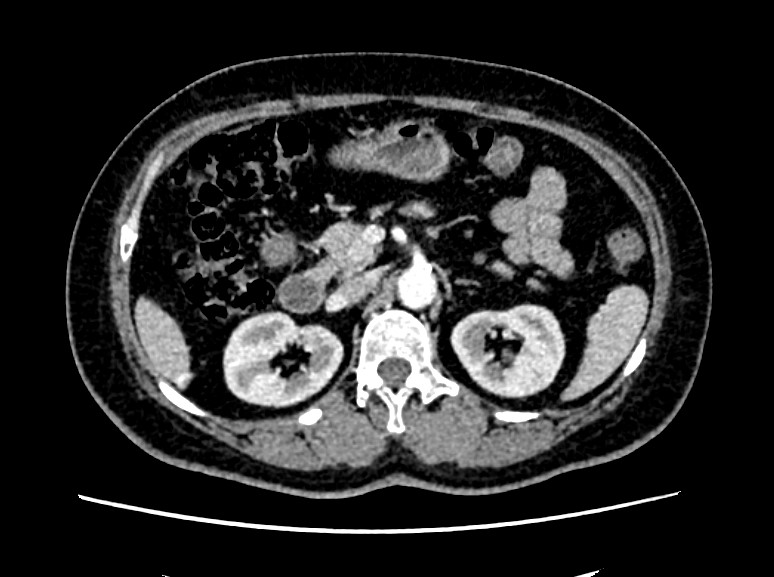

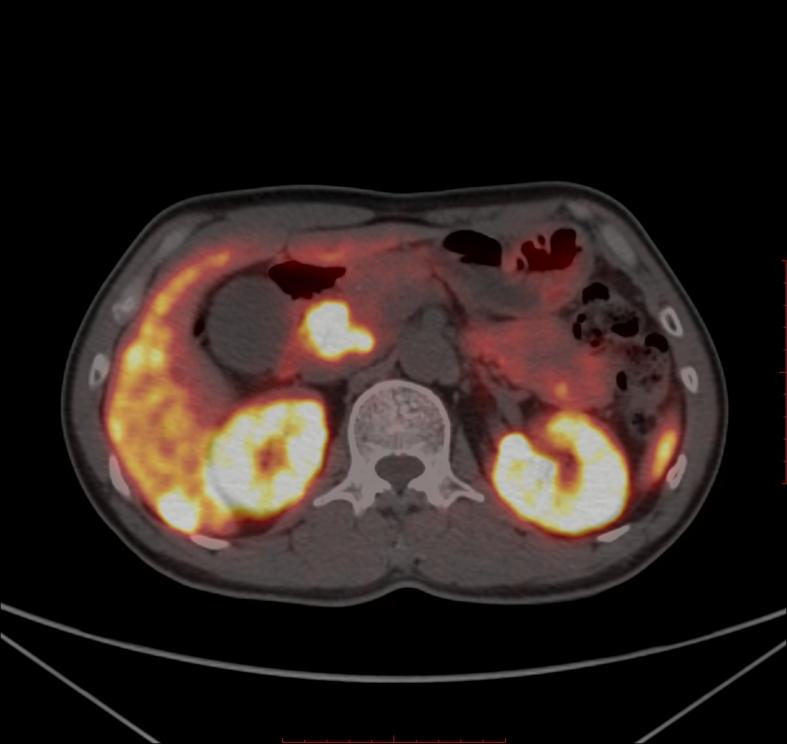

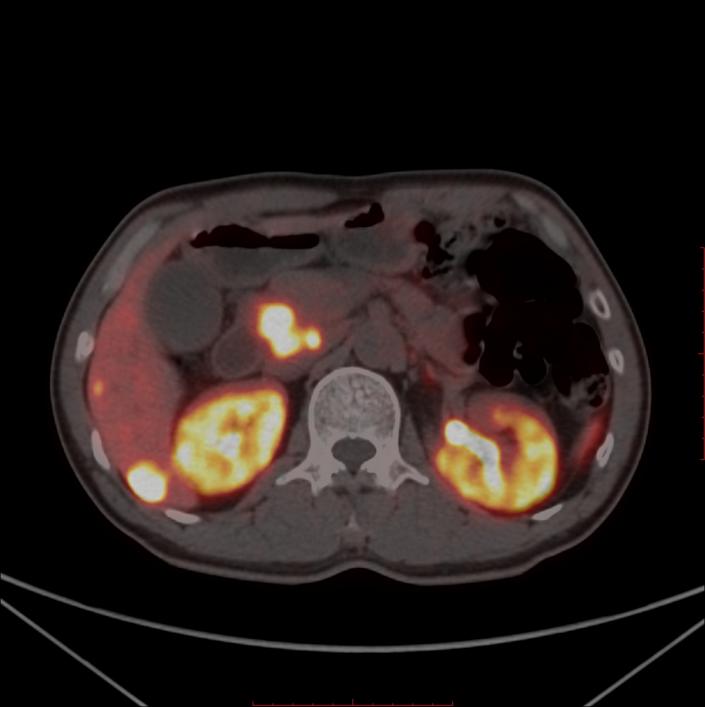

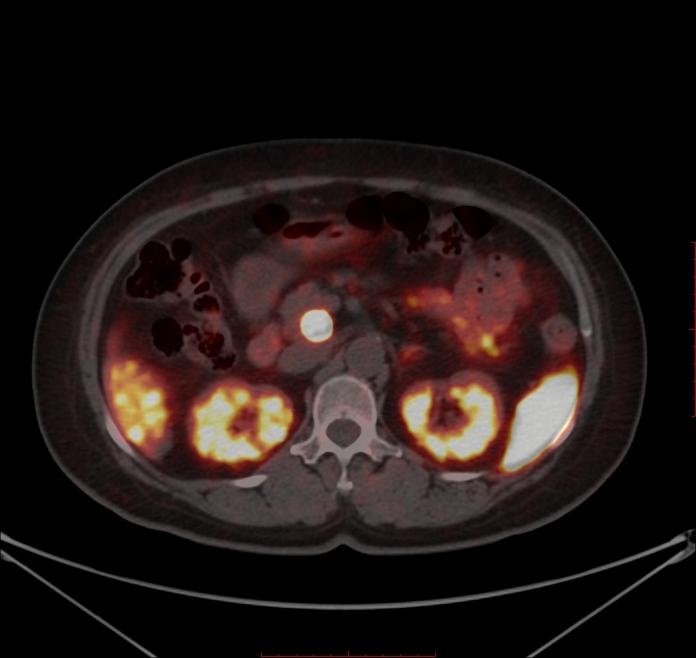

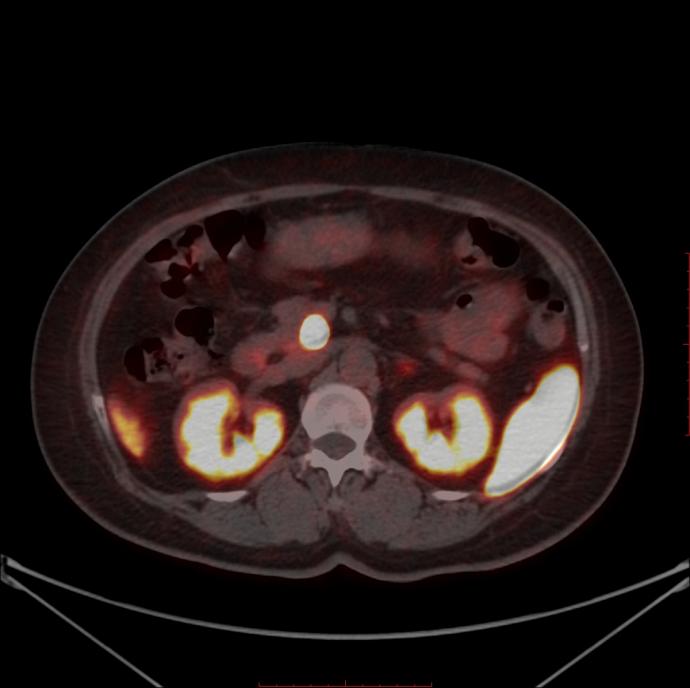

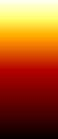

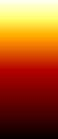

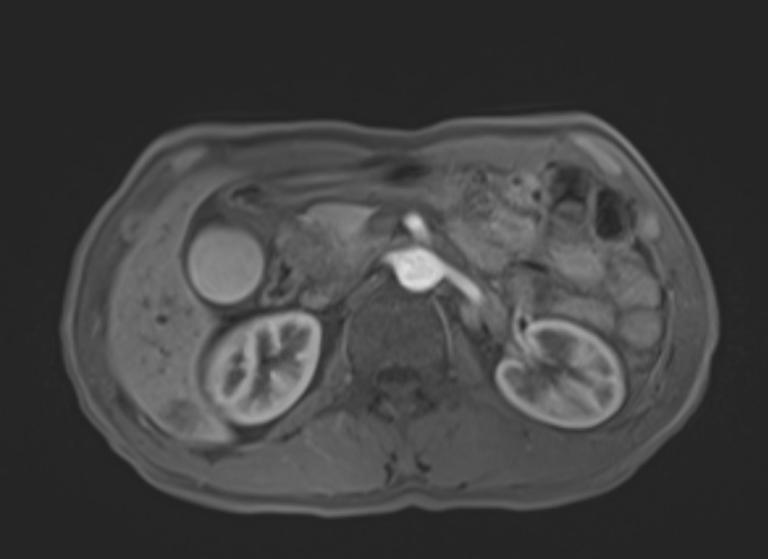


CT

MRI

a-c: A 50-year-old male patient with a G2 NEN of the PU (red arrow) with liver metastases (white arrow). Both SST-positive lesions show contrast enhancement on the MRI image (a ^68^Ga-DOTATATE PET/CT fused image, b diagnostic MRI image, c ^18^F-OC PET/CT fused image). The SUV_max_ on ^68^Ga-DOTATATE and ^18^F-OC of PU are 16.4 and 15.3, respectively.

d-f: A 56-year-old female patient with G1 NEN of the PU (red arrow). An abnormal enhancement of PU found on the CT (e) shows abnormal uptake in both ^68^Ga-DOTATATE PET/CT fused image (d) and ^18^F-OC PET/CT fused image (f), with the SUV_max_ of 86.7 and 94.3, respectively.

10

0

10

^68^Ga-DOTATATE

e

a

^68^Ga-DOTATATE

^18^F-OC

0

^18^F-OC

b

c

d

f
